# 

**Published:** 1815-07

**Authors:** 


### THE LONDON MEDICAL AND PHYSICAL JOURNAL;

CONTAINING

ORIGINAL CORRESPONDENCE of EMINENT PRACTITIONERS,

AND THE

EARLIEST INFORMATION ON SUBJECTS CONNECTED WITH

MEDICINE, SURGERY, CHEMISTRY, PHARMACY, BOTANY, AND NATURAL HISTORY.

*Successively conducted by*

T. BRADLEY, M.D.

A. F. M. WILLICH, M.D.
R. BATTY, M.D.
A. A. NOEHDEN, M.D.
J. ARNEMAN, M.D.
J. P. PFAFF, M.D.
J. ADAMS, M.D.
W. SHEARMAN, M.D.
W. ROYSTON, Esq.

AND

SAMUEL FOTHERGILL, M.D.

Assisted by eminent Gentlemen, in the various Branches of the Profession.

5140

VOL. XXXIV.

FROM JULY TO DECEMBER, 1815.

Et quoniam variant morbi, variabimus artes;
Mille mali species, mille salutis erunt.

*LONDON:*

PRINTED FOR THE PROPRIETORS, BY J. ADLARD, 23, BARTHOLOMEW-CLOSE, AND 39, DUKE-STREET, SMITHFIELD.

PUBLISHED BY J. SOUTER, NO. 1, PATERNOSTER-ROW; AND MAY BE HAD OF ALL BOOKSELLERS.

W1
LO 31

[Entered at Stationers' Hall.]

### MONTHLY CATALOGUE OF MEDICAL BOOKS.

THE Hunterian Oration, delivered in the Theatre of the Royal College of Surgeons, on the 14th Day of February, 1815. 4to. Rivington.

A Practical Treatise and Observations on the Nature, Variety, and Treatment, of the Venereal Disease, &c. &c. By F. Kiernan. Highley and Son.

Pharmacopœia and Materia Medica, composed for the Use of young Physicians, and especially intended to accompany the Pathological System of Medicine. By John Bell.

A Logometric Scale of Agricultural Equivalents. By the Penrith Agricultural Society. Callow.

An Introduction to Entomology: or Elements of the Natural History of Insects: with Plates. By William Kirby, B.A. F.L.S. and William Spence, Esq. F.L.S. Vol. I. Longman and Co.

Conspectus Medicinæ Theoreticæ, ad Usum Academicum.—Auctore Jacobo Gregory, M.D. Longman and Co.

Practical Observations on the Treatment of Gleets and Strictures of the Urethra. By John Andree, 8vo. Callow.

A popular Treatise on the Venereal Disease, in which is exhibited all the recent Discoveries: and a certain Cure for that terrible Malady. By Robert John Thornton, M.D. 12mo. Cox and Son.

---

### *NOTICES TO CORRESPONDENTS.*

*The increased applications for an Index to the first Thirty-three Volumes of the London Medical and Physical Journal is at length so considerable, as to induce the Proprietors to commence some arrangements for its accomplishment. It is extremely difficult to calculate the numbers likely to be wanted. The Subscribers, during fifteen years, must have been fluctuating, especially as so many numbers have been required for the professional gentlemen in both the services. It is therefore requested, that such of their Correspondents, as wish for a Copy, will send their names, as very few additional numbers will be printed.*

*Communications are received from Drs. Sutton, Kinglake, and several other Correspondents, which will appear in our next.*

